# Supplementary material for: CLigOpt: controllable ligand design through target-specific optimization
Source: Bioinformatics. 2024 Sep 4;40(Suppl 2):ii62–9. doi: 10.1093/bioinformatics/btae396 (PMC11373314; doi:10.1093/bioinformatics/btae396)
Supplement: btae396_Supplementary_Data [file btae396_supplementary_data.zip › CLigOpt_ECCB2024_184_Li_etal_suppl.pdf]

*Bioinformatics*, 2024, pp. 1–1

doi: DOI HERE
ECCB 2024

ECCB 2024

# CLigOpt: Controllable Ligand Design through Target-Specific Optimisation

Yutong Li,[1] Pedro Henrique da Costa Avelar,[1,2] Xinyue Chen,[1] Li Zhang,[2] Min Wu[2] and Sophia Tsoka[1,*]

[1]Department of Informatics, King's College London, Bush House, WC2B 4BG, London, UK and [2]Institute for Infocomm Research, Agency for Science, Technology and Research (A*STAR), 1 Fusionopolis Way Connexis Tower, 138632, Singapore
[*]Corresponding author. sophia.tsoka@kcl.ac.uk

## Abstract

**Algorithm S1** CLigOptCensNet: Training

**Input** Fragment graphs $G_F$, Molecular graphs $G_M$, and generation condition $c$
**Output** Edge probabilities $edge\_probs$, and $edge\_type\_probs$, and node probabilities $node\_probs$

```
emb_F, emb_M ← Embedding(G_F), Embedding(G_M)          ▷ Encoder
H_F, H_M ← CensNet(emb_F), CensNet(emb_M)
H_MG ← GAP(H_M, batch)
μ_F, μ_M ← NNμ(H_F), NNμ(H_M), log(σ)_F, log(σ)_M ← NN_σ(H_F), NN_σ(H_M)
z_M ~ N(μ_M, σ_M), noise ~ N(0, 1)    ▷ Adding fragment attention to the initial graph sample
z_F ← Add(μ_F, noise)
a_F ← Attention(z_F)
z ← z_F + a_F * z_M
z ← [z, H_M]
for t in steps do                                       ▷ Decoder
    H^(t) ← CenNet(H^(t−1))
    edge_probs^(t), edge_type_probs^(t) ← NN_e(H^(t)), NN_et(H^(t))
end for
a_N ← Attention(z)                                      ▷ Node Prediction
H_N ← NN(z)
z_M ← H_N + a_N * z
node_probs ← NN_node(z_M)
```

**Algorithm S2** CLigOptCensNet: Generation

**Input** Fragment graphs $G_F$, and generation condition $c$
**Output** Result Molecule $mol$

```
emb_F ← Embedding(G_F)
H_F ← CensNet(emb_F)
μ_F ← NNμ(H_F), log(σ)_F ← NN_σ(H_F)
z_M ~ GM(z), noise ~ N(0, 1)
z_F ← Add(μ_F, noise)
a_F ← Attention(z_F)
z ← z_F + a_F * z_M
accept ← CLaSS(z)
if not accept then
    draw a new z_M
end if
z ← [z, H_M]
edge_probs, edge_type_probs, node_probs ← NN_e(z), NN_et(z), NN_node(z)
mol ← assign edge_probs, edge_type_probs, node_probs
```

© The Author 2024. Published by Oxford University Press. All rights reserved. For permissions, please e-mail: journals.permissions@oup.com

| Model | ZINC | | | | CASF | | | |
|---|---|---|---|---|---|---|---|---|
| | DeLinker | FFLOM | CLigOptGCN | CLigOptCensNet | DeLinker | FFLOM | CLigOptGCN | CLigOptCensNet |
| Validity | 99.33% | 100.00% | 100.00% | 98.02% | 98.00% | 100.00% | 100% | 99.38% |
| Uniqueness | 41.23% | 95.81% | 54.49% | 71.44% | 15.41% | 96.10% | 64.44% | 73.83% |
| Novelty | 37.32% | 99.00% | 72.44% | 86.76% | 41.03% | 99.21% | 77.46% | 89.59% |
| SA filter | 95.93% | 25.83% | 89.03% | 85.21% | 92.33% | 17.70% | 72.05% | 67.04% |
| Ring Aromaticity | 92.90% | 97.15% | 85.17% | 93.97% | 90.83% | 94.99% | 73.83% | 82.09% |
| PAINS filter | 98.32% | 94.14% | 98.20% | 98.12% | 99.41% | 92.85% | 97.33% | 97.92% |
| Average | 77.51% | 85.32% | 83.22% | **88.92%** | 72.84% | 83.48% | 80.85% | **84.98%** |

**Table S1.** Results for the two baselines (DeLinker and FFLOM) and our two models (CLigOpt{GCN,CensNet} for each of our evaluated metrics, as well as the average result along all models, on our two datasets (ZINC and CASF). We see that our CensNet model has the best average performance on both datasets.

| | QED>0.4 | QED>0.5 | QED>0.6 | QED>0.7 |
|---|---|---|---|---|
| CLigOptGCN | | | | |
| Random | 21.27% | 14.68% | 9.78% | 4.84% |
| Accepted | 73.75% | 63.99% | 57.71% | 28.91% |
| CLigOptCensNet | | | | |
| Random | 25.16% | 16.65% | 11.17% | 6.17% |
| Accepted | 55.26% | 52.00% | 49.47% | 28.09% |

**Table S2.** The proportion of sampled molecules that passed the QED filters for our models when sampling randomly versus when sampling using the CLaSS accepted set.

| | SA<3 | SA<2.5 |
|---|---|---|
| CLigOptGCN | | |
| Random | 16.27% | 2.66% |
| Accepted | 31.58% | 13.81% |
| CLigOptCensNet | | |
| Random | 13.23% | 3.99% |
| Accepted | 22.07% | 12.08% |

**Table S3.** The proportion of sampled molecules that passed the SA filters for our models when sampling randomly versus when sampling using the CLaSS accepted set.

| | pIC50>6 | pIC50>6.5 | pIC50>7 |
|---|---|---|---|
| CLigOptGCN | | | |
| Random | 53.32% | 24.27% | 11.49% |
| Accepted | 58.49% | 26.86% | 12.34% |
| CLigOptCensNet | | | |
| Random | 56.99% | 26.39% | 11.08% |
| Accepted | 62.78% | 29.02% | 12.17% |

**Table S4.** The proportion of sampled molecules that passed the pIC50 filters for our models when sampling randomly versus when sampling using the CLaSS accepted set.

| | Transformer-CNN | modSAR | DeepAffinity |
|---|---|---|---|
| RMSE | 0.8160 | 1.6902 | 1.7138 |

**Table S5.** Binding Affinity Predictor

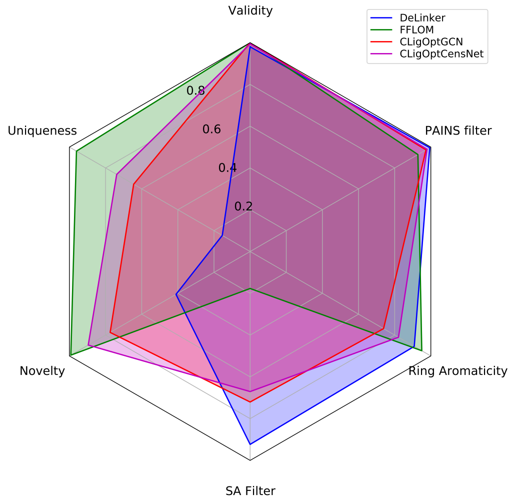


**Fig. S1.** A radar plot showing the performance of our model as well as the two baselines on CASF. Although our model does not outperform the best model in each category, CLigOptCensNet achieves the best average performance.

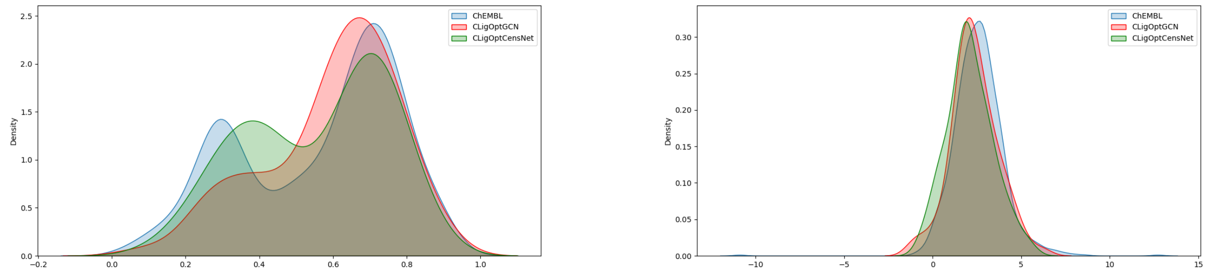


(a) The QED distribution of hDHFR inhibitors, molecule sets generated by CLigOptGCN, and CLigOptCensNet.

(b) The LogP distribution of hDHFR inhibitors, molecule sets generated by CLigOptGCN, and CLigOptCensNet.

**Fig. S2.** The distribution of comparing attributes of generated sets and hDHFR inhibitors.
